# Pediatric Autism Diagnosis Accuracy and Confidence: A Comparison of Experienced and Inexperienced Clinicians Making Decisions with and without AI Decision Support

**DOI:** 10.21203/rs.3.rs-9546356/v1

**Published:** 2026-06-24

**Authors:** Gondy Leroy, Sumi Lee, Krishna Prashanth Thummanapelly, Winslow Burleson, Nell Maltman, Sydney Rice, Joshua Rothman

**Affiliations:** 1 Management Information Systems, University of Arizona, Tucson, AZ, USA; 2 Linguistics, University of Arizona, Tucson, AZ, USA; 3 Computer Science, University of Arizona, Tucson, AZ, USA; 4 Information Science, University of Arizona, Tucson, AZ, USA; 5 Speech, Language, and Hearing, University of Arizona, Tucson, AZ, USA; 6 Pediatrics, University of Arizona, Tucson, AZ, USA; 7 Pediatrics, University of California, San Diego, CA, USA

**Keywords:** autism, ASD, artificial intelligence, AI, decision support, automation bias

## Abstract

**Background:**

Autism Spectrum Disorders (ASD) is a neurodevelopmental condition where early diagnosis is extremely important for optimal treatment effects. Unfortunately, the current age of diagnosis is made late due to a variety of factors, including a lack of clinicians with ASD expertise. We developed Autism Diagnostic Identification Software (ADIS), an AI-based clinical decision support tool that identifies autism-relevant behaviors in narrative clinical text and labels them with DSM5 diagnostic criteria. Using current DSM5 rules, an autism diagnosis is then suggested. Our aim is to provide pre-decision support, which differs from explainable AI approaches, in which AI decisions are clarified post hoc.

**Methods.:**

To evaluate the impact of AI suggestions, we conducted a vignette-based user study with 21 clinicians (48% with completed medical training), each of whom reviewed four real pediatric cases (two with ADIS support and two without), resulting in 84 diagnostic decisions. The cases were chosen so that ADIS also suggested correct and incorrect decisions to study participants.

**ResuIts:**

Overall diagnostic accuracy was 57.14% when ADIS was active versus 66.67% without ADIS, a nonsignificant difference. Decision confidence was higher with ADIS (F(1,80) = 3.71, p = .058), and significantly higher among clinicians who had completed their medical training (F(1,80) = 18.45, p < .001). There was also significant interaction between ADIS correctness and training completion (F(1,38) = 5.05, p = .031): trainees showed 0% accuracy when ADIS was wrong versus 68.75% when it was correct, whereas trained clinicians achieved 66.67% and 56.25% accuracy in those conditions, respectively. Despite these differences in accuracy, confidence was high regardless of ADIS correctness. Most participants (90%) reported that ADIS use was learned quickly and rated its features as well integrated.

**Conclusions:**

These findings suggest that AI support can help inexperienced clinicians, but also highlight the importance of AI accuracy, given the observed overreliance on AI. ADIS is usable and perceived as helpful, but its deployment will be most effective when paired with AI literacy training that mitigates automation bias.

## 1. Introduction

Autism spectrum disorder (ASD) is a neurodevelopmental condition characterized by persistent differences in social communication and interaction, alongside restricted and repetitive behaviors. Early diagnosis is associated with improved access to intervention and better long-term outcomes, yet the average age of diagnosis remains substantially later than the age at which reliable identification is possible.

Recent advances in artificial intelligence (AI) have generated substantial interest in improving ASD screening and diagnosis. Machine learning approaches have demonstrated the ability to identify autism-related behavioral patterns. However, most AI-based ASD research has focused on algorithmic accuracy under controlled conditions, with little attention to clinicians’ experience and use of such systems in practice. In clinical contexts, the utility of AI systems depends not only on performance metrics but also on interpretability, workflow compatibility, and appropriate trust calibration. With the increasing availability of AI tools, it has become more important to evaluate how these tools are integrated into the decision-making process and their effects on clinical decisions. This is especially true since few clinical users are AI specialists, and AI tools are rarely 100% accurate. Relying on AI decisions may improve, augment, or worsen decision quality.

The study addresses this gap by evaluating ADIS, which provides pre-decision AI support that highlights autism-relevant behaviors. Rather than producing a standalone diagnosis, ADIS supports clinician recognition of ASD-indicative behaviors that may warrant further evaluation. In this work, we report findings from a user study examining diagnostic accuracy and decision confidence among expert and non-expert users, and ADIS usability.

## 2. Background

### 2.1. AI in ASD Screening and Diagnosis

Although reliable identification of ASD is possible in early childhood, many individuals experience substantial delays between the appearance of the first concern and a formal diagnosis. These delays are due to the complexity of ASD presentation, limited access to specialists, and fragmented clinical information over time. AI is increasingly being developed for the use of ASD screening and diagnosis. Several projects have focused on applying supervised machine learning to behavioral assessment data. In many cases, multimodal machine learning approaches integrate behavioral, developmental, and physiological data, and focus on multiple sources of information across the diagnostic workflow to support different stages of ASD evaluation (1, 2). Such cross-domain classifiers have been used with early behavioral and developmental measures to identify ASD very early, e.g., at age three in high-risk infants (3).

Other work emphasizes feature selection and data size. For example, machine learning models have been used to identify robust behavioral predictors of ASD (4), addressing concerns about redundancy and overfitting. Computational methods have also been shown to detect autism-related behavioral patterns using reduced sets of screening features, suggesting that efficient screening could be achieved without full-length diagnostic instruments (5). Focusing on Autism Diagnostic Observation Schedule (ADOS) data, feature selection techniques have shown that small subsets of behaviors retained strong discriminative power between ASD and non-ASD cases (6). Complementary approaches focused on the number of required observations and demonstrated that machine learning could substantially reduce the number of behavioral observations needed for accurate classification, highlighting the potential to shorten diagnostic timelines while preserving clinical relevance (7). Collectively, these studies establish that AI systems can identify meaningful behavioral signals associated with ASD.

As AI methods matured, attention increasingly turned toward clinical validation and integration in the workflow. A large-scale crowdsourced validated machine learning classifiers that distinguished between ASD and ADHD, addressing concerns about scalability and generalizability (7), which is a critical challenge in real-world diagnostic contexts. EHR-based algorithms have been developed to retrospectively identify ASD cases at scale (8–11). EHR-driven tools have also been shown to improve feasibility and efficiency in autism identification in clinical workflows (12). Outside of EHR integration, an AI-based medical device for ASD diagnosis was shown to provide the necessary evidence for regulatory approval and clinical deployment (13). With increased clinical use, usability and interface design are ever more important and require clinician feedback to improve the functionality and acceptability of digital ASD screening tools. (14). These studies marked important steps toward translating AI systems from research prototypes into clinically viable tools.

### 2.2 Impact of ArtificiaI Intelligence on Medical Decision Making

Human decision-making supported by algorithm-generated recommendations is not new. As algorithms, especially AI, grow more sophisticated and as they are increasingly embedded in everyday life, their impact on our decision-making for health and well-being increases too.

In early work, contrasting findings on AI use in decision-making were reported. Some studies documented algorithm aversion, where humans preferred human decisions even when algorithmic decisions were shown to be better (15), which was challenged by a set of studies across different domains where algorithmic decisions were preferred by non-experts but not experts (16). Beyond this dichotomy, recent clinical trials have identified a persistent automation bias, wherein clinicians over-rely on automated outputs even when they contradict direct clinical observation. This bias represents a significant risk of harm when AI systems are deployed as assistive tools without sufficient human-centric validation (17). Over-reliance is often exacerbated in high-acuity settings where cognitive load is high, potentially leading practitioners to use algorithmic outputs as a heuristic rather than a consultative data point (18).

Since even the best AI models will not be perfect, understanding their weaknesses is needed (19). For example, training to recognize algorithmic bias (the risk that models trained on biased datasets will perpetuate health inequities among marginalized populations) or data drift (where a model’s performance degrades over time as patient demographics or clinical guidelines evolve) can improve the appropriate use of AI in the workflow (20). However, decision weaknesses are amplified when the AI decision process is unclear or not understood. For example, black-box AI tools have been shown to increase underdiagnosis of female and minority patients, whose presentations deviate from the predominantly male, white samples on which most diagnostic models were trained (21, 22). Furthermore, automation bias (over-reliance on automated recommendations) is worse when domain knowledge is reduced. For example, certified training and physician-level experience significantly reduced false agreement with incorrect AI recommendations; however, it did not eliminate automation bias entirely (23). Medical trainees, who have less training and experience, have been identified as a particularly high-risk group for uncritical acceptance of AI (24). Furthermore, newer AI systems rely on opaque processes and use generative AI to communicate outcomes in a human-like manner, thereby further obscuring the underlying algorithmic process.

These developments highlight the dangers of black-box AI and the additional factors that must be considered when designing and evaluating such systems (16). Some emphasize that AI systems must only support clinician reasoning rather than replace it (25, 26). In other cases, explanation of decisions is emphasized, and here, Explainable AI (XAI) frameworks play a major role in guiding this development. These frameworks provide model-agnostic explanations post-decision, allowing the clinician to see which variables (e.g., age, specific biomarkers, or co-morbidities) most heavily influenced the AI’s output (27). Integrating XAI frameworks has been advocated as essential to ensure these systems augment rather than distort assessment (20, 27, 28). Interestingly, such explanations can further complicate confidence in a decision, i.e., they have been shown to increase clinician confidence without improving accuracy (29). In response to this new complication, it has been shown that explicit uncertainty cues alongside AI predictions can improve appropriate trust calibration (30), suggesting that communicating model uncertainty is a tractable design intervention.

### 2.3 Study Rationale and Research Questions

While AI continues to improve rapidly, collaboration between AI and clinical experts requires attention, since few clinical experts are trained in AI. User studies that evaluate interpretability, workflow integration, and trust calibration are essential for understanding the real-world value of AI in ASD care. Our primary research question focuses on the extent to which AI can support ASD diagnosis by providing information before a diagnostic decision, rather than post-decision explanations. We focus primarily on decision accuracy and confidence, as well as the quality of the documentation supporting these. We explicitly evaluate the impact of an incorrect AI diagnosis across different levels of clinical expertise.

## 3. Autism Diagnostic Identification Software (ADIS)

Our Autism Diagnostic Identification Software (ADIS) is a clinical decision support system designed to assist clinicians in identifying autism-relevant behaviors as they are described in clinical and lay texts. In this study, we focus on clinical text, which applies to situations in which new patient information is received, a longitudinal review is needed, or the pediatrician is not an ASD expert.

ADIS was designed to highlight information relevant to diagnosis and to support transparency, reproducibility, and clinician interpretability by providing behavioral classifications linked to DSM5-aligned categories. The underlying BioBERT model was trained on clinical and lay (e.g., parental) behavioral descriptions, and it labels text snippets with one or multiple of the seven DSM5 criteria (31). For each label, the model assigns a probability that can be used to apply more or less strict labeling. For example, a 30% probability of a behavior meeting the A2 criterion could be accepted by a lenient model; a strict model may require a probability of 70% or higher. While tagging individual behaviors is not 100% accurate, the case-based sensitivity and specificity of diagnosing ASD are comparable to those of human experts due to redundancy in labeled behaviors (31, 32).

**Figure 1:**
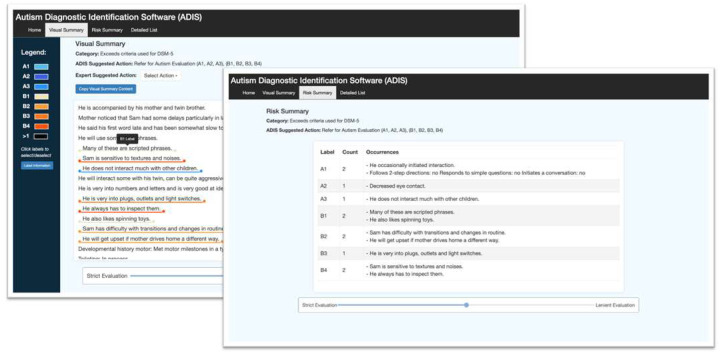
ADIS Interface

The ADIS interface (shown in Figure 1) shows the home page, which allows narrative text input. Upon submission of clinical text, the back-end BioBERT algorithm segments the text into sentences and applies the model to each sentence to assign the labels or to indicate that no clinical behavior was present. The tagged text is then displayed in different ways.

The first results tab, Visual Summary, is interactive: the original text is shown with one sentence per line, and those sentences labeled with a DSM5 criterion are underlined in different colors. Users can turn underlining on and off using the legend on the left. For example, Figure 1 shows that the sentence “He does not interact much with other children” is underlined to indicate that the A1 DSM5 label was assigned. In addition to the interactive tab, ADIS includes two static overviews that display case details. The Risk Summary tab shows a summary by DSM5 criteria; for example, there are two text snippets labeled as A1 behaviors. The Detailed List tab shows the complete list with all text, one sentence per line, and shows the label if one was assigned. This tab is not interactive and does not use color underlining.

A final suggestion for an autism decision is also stated in a section above the listed behaviors. This final suggestion relies entirely on the number of behaviors labeled and applies current DSM5 rules, i.e., the number of A and B criterion behaviors that must be present for a diagnosis. The number of A and B criteria that were matched in the text is reported along with this decision suggestion.

The user can move a slider to adjust the strictness of label matching. The slider sets the minimum threshold probability that the BioBERT model must have assigned; i.e., the stricter the model, the higher the model’s probability for this label must be to attach it. With a strict evaluation, fewer behaviors are usually labeled with a DSM5 label, and with a lenient evaluation, more are usually labeled, which affects the suggested diagnosis. No probability numbers are shown in the interface; however, moving the slides dynamically updates the interface labels and the associated suggested diagnosis.

## 4. Methods

### 4.1 Overview

Our study used an online, vignette-based design to evaluate AI-based decision support. In this context, a vignette was a case description of a child to be evaluated for autism. The objectives were to assess the tool’s usefulness in supporting decision-making and to examine how AI-generated suggestions influenced this decision-making process among users with and without completed medical training (i.e., expert and novice evaluators).

For the purposes of the user study, the input text field was automatically populated with the case text when study participants selected their assigned case from a drop-down list. This drop-down list was added to the interface for the study only to ensure identical, fast case presentations for all participants, regardless of case length.

### 4.2 Cases

We selected cases from the University of Arizona’s Clinical Data Warehouse (CDW) and from the Autism and Developmental Disabilities Monitoring (ADDM) Network from the Centers for Disease Control and Prevention (CDC). We chose cases that reflect different types of records that a pediatrician may receive.

For the study, all information was combined per child and de-identified. In some cases, information about the DSM5 labels themselves (i.e., their definitions) was included in the original narrative and was removed for the study. We chose to work with real cases to enhance the realism of our study and to reflect the extent of available information. We selected cases to achieve a balanced representation of records with varying numbers of clinically relevant behaviors (low, medium, high), gender (male, female), and final diagnosis assigned by an expert (autism or not), as well as accurate and inaccurate machine labeling and suggestions (See Appendix A).

The ASD human expert diagnosis of the child (ASD or No ASD) and the diagnostic labels (A1-B4) used to select the cases were originally assigned by our CDC-trained labelers during algorithm training and reused for this study. The cases themselves were not included in the original algorithm training, i.e., they were part of the test set. The human expert review was necessary at that time because the ADDM records had final labels, whereas the EHRs were coded using ICD-10 codes, which do not always align with diagnoses. This information was not shown to the participants.

While not every possible combination of variables could be tested, we controlled the following variables:

- Gender of the child: male or female
- Number of significant behaviors mentioned in the case: high, medium, or low. These are the number of behaviors flagged as a DSM diagnostic criterion. We accounted for the combined number of flagged behaviors (i.e., no distinction by individual criteria) and divided the cases into the top 30%, bottom 30%, and the remaining middle section.
- The correctness of the ADIS diagnosis: correct or incorrect.

Using these criteria, we selected eight cases for use in the study. We named each child in the case with a gender neutral name: Sam, Lee, Ashley, River, Bin, Taylor, and Obany. Appendix A shows the details of the cases and Table 1 show the case overview. Even though ADIS has previously demonstrated high sensitivity and specificity comparable to a human expert (32)We included cases in this study in which ADIS’s initial decision was incorrect. This allowed us to evaluate whether and how easily users might detect, correct, or propagate incorrect AI-supported suggestions. Participants were not informed about the correctness of ADIS.

### 4.3 Participants

We invited pediatricians to participate by email through our professional network. The email was sent out to clinicians and medical students at the University of Arizona and the University of California, San Diego. Those who were interested received the link to the study site and an additional explanation. Our goal was to have pediatricians with different backgrounds. None of the pediatricians had knowledge of our system or its components, or any formal education in AI. Twenty-two clinicians (N = 22) participated and completed all study procedures.

All participants completed the study remotely, reviewing cases using the ADIS interface and providing structured and open-ended feedback. No personally identifiable patient information was used, and the study focused exclusively on clinician interaction with the system rather than patient outcomes.

### 4.4. Study Procedure

#### Invitation and Consent:

Pediatricians who consented to participate in the study were invited via email and given a codename, which they used on Qualtrics. Qualtrics allowed us to control the order of case presentation and to record participant responses systematically. Participants were informed that the study involved a review of four clinical cases, two of which were presented with ADIS support and two without.

#### Welcome and Instructions:

First, participants were shown a page that served as both a welcome and an informed consent page (approved by the Institutional Review Board (IRB) of the University of Arizona). Here, they were also provided with an overview of the study purpose, which was to evaluate a newly developed software system designed to support recognition of ASD from electronic health record (EHR) text. It was explained that they would review aggregated EHR notes for pediatric cases and were asked to indicate whether, based on the available information, they would consider a referral for an autism evaluation. They were instructed to complete the study in the order presented and were advised not to use a mobile device. The instructions indicated that participants were not required to consult external resources but were permitted to do so if desired.

Then, participants were asked to complete demographic questions (see Appendix A) covering race, ethnicity, gender, and professional experience questions.

#### Case Grouping:

Each participant was assigned four cases, which were arranged so that different participant groups were exposed to varying levels of model correctness. For two of those cases, ADIS aided in DSM criteria labeling. The order of four cases in each group was randomized. Limiting the number of cases helps avoid fatigue or boredom. For River and Obany, ADIS made the wrong decision when it was active.

The four groups were as follows (not yet randomized):

- Group 1: River (ADIS) - Bin (ADIS) - Lee (No ADIS) - Blue (No ADIS)
- Group 2: Ashley (ADIS) - Obany (ADIS) - Sam (No ADIS) - Tyler (No ADIS)
- Group 3: Sam (ADIS) - Tyler (ADIS) - Ashley (No ADIS) - Obany (No ADIS)
- Group 4: Lee (ADIS) - Blue (ADIS) - River (No ADIS) - Bin (No ADIS)

For each group, a separate Qualtrics survey was created with the four cases and a link for the participants to follow. This allowed us to control whether ADIS was active and the order of the cases. Naturally, the evaluation questions were always the same.

#### Case Review Pages:

On the ADIS interface, participants selected the case from a dropdown menu, which loaded the relevant information. When the tool’s functionality was enabled, key behaviors identified by the underlying algorithms were underlined, and a suggested diagnosis was provided. Participants could navigate between detailed tables and summary views. When the functionality was disabled, the text was displayed without any augmentation. After reviewing each case, participants returned to the Qualtrics survey to submit their evaluation and answer the case questions (See Table 2).

After completing the four cases, participants were invited to provide a qualitative evaluation of the tool as well as comments or suggestions for tool improvement (See Appendix B).

#### Study Conclusion:

Upon completion of the study tasks, participants were asked to provide contact information. Contact information was used for reimbursement purposes via an Amazon gift card and was not linked to study responses.

## 5. Results

### 5.1 Demographics

A total of 23 individuals initiated the study; however, two participants did not complete the cases in the correct order, as indicated by the confirmation question, and their data were excluded. The study sample (Table 3) comprised 21 participants, most of whom were between 20 and 40 years of age (81%), with the remainder aged 41–50 (10%) and 51 years or older (10%). The majority identified as female (86%), with 9% identifying as male and 5% as non-binary. In terms of race, 62% identified as White, 14% as Asian, and smaller proportions as Black or African American (5%), Native Hawaiian or Other Pacific Islander (5%), Middle Eastern or North African (5%)(MENA), or another racial background, with 5% preferring not to answer. Overall, 24% of participants identified as Hispanic or Latino. Just under half of the sample reported having completed medical training (48%), while 52% did not, yielding a mix of participants with and without formal clinical training.

Participants represented a range of clinical roles, including practicing clinicians and trainees in medicine and pediatrics (See Appendix C for details). Based on their self-report, the sample included individuals currently in medical school, pediatric residency or fellowship training (including developmental-behavioral pediatrics), and practicing providers involved in pediatric care. This heterogeneous sample was intentionally recruited to reflect the range of clinicians who may encounter early autism-related concerns in clinical workflows.

### 5.2 Case decisions

We first provide the accuracy of decisions and compare those where ADIS was active or not and for participants with and without medical degree completion (Table 4). Overall, we found that with ADIS active, the accuracy of decision-making was 57.14% and without ADIS active it was 66.67%. When taking completion of education into account, those who completed their medical degree were 63.64% accurate and those who had not yet completed it were 60.00% accurate. A two-way ANOVA indicated no significant main or interaction effect.

For each decision, we also request the confidence that the participants had in their decision (Table 5). We recoded “Not Confident” as zero, “Confident” as one, and “Very Confident” as two. We found that when ADIS was active, confidence was on average 1.33 while it was 1.09 when it was not active. Our two-way ANOVA shows this to be strong trend (F(1,80)=3.713, p =0.58). Participants who had completed their medical training reported an average confidence of 1.48 and those who did not yet complete 0.92, which was statistically significant (F(1,80)=18.454, p <.001). The interaction was not significant.

In a second analysis (Table 6), we evaluated the results for those cases where ADIS was active. Overall, we see that accuracy drops to 40.0% when ADIS suggested an incorrect decision compared to 62.5% when ADIS suggested a correct decision. When taking degree completion into account, we see that the accuracy of the decision is 0% for those who did not complete their degree when ADIS suggested the wrong decision and 68.75% when ADIS suggested the correct decision. For those who completed their degree, accuracy was 66.67% when ADIS suggested an incorrect decision and 56.25% when ADIS suggested the correct decision. A two-way ANOVA showed this interaction effect to be significant (F(1,38)=5.049, p = 0.031). There were no significant main effects.

Participants with and without a completed medical degree show similar high confidence, i.e., 1.50 and 1.15, respectively. Interestingly, confidence is also high regardless of the correctness of ADIS with an average score of 1.40 when ADIS gave a wrong suggestion and 1.31 when the suggestion was correct (Table 7). Our two-way ANOVA showed no significant main or interaction effect for confidence.

### 5.3 Usability Analysis

After completing their four cases, participants were requested to complete the usability survey (Table 8). Overall, participants reported a positive experience with ADIS, with most agreeing that they would use it frequently and that its features are well integrated. A large majority (about 90%) felt that most people would learn to use the tool very quickly, which was the highest-rated item, and most respondents expressed confidence in using the tool. Furthermore, nearly two-thirds disagreed that they needed to learn a lot. Even so, since one-quarter agreed it would take learning, there is room to add initial guidance. Importantly, very few participants considered the tool complex, inconsistent, or cumbersome, reinforcing the generally favorable perception of its usability.

We also asked about further use of ADIS using three multiple-choice questions (Table 9) and two open-ended questions. Most participants indicated that they would like additional functionality, particularly the ability to download results in a document format and to have the tool automatically evaluate all records at their facility (both endorsed by about 71% of respondents). Just over half (about 52%) wanted to forward results via email, while the remainder were split between preferring not to or being unsure, suggesting some variability in preferences for sharing workflows.

The open-ended questions provide an opportunity to highlight strengths and weaknesses. Some participants requested a clearer linkage between the tool’s outputs and DSM5 criteria, as well as identification of non-autism-associated features (7 respondents). A smaller group (4 respondents) emphasized the need for better guidance, such as tutorial videos, clearer explanations of labels like A-B4, and the strict versus lenient settings. Others (5 respondents) focused on usability, including improving highlighting, making the detailed lists less cumbersome, and allowing users to organize information or provide feedback to the tool. Individual suggestions included calculating an explicit likelihood of referral need, integrating current problem lists and medications from the chart, adding a simple DSM5 criteria view, and (one) addressing a technical issue (the need to refresh due to a case disappearing after clicking evaluate).

Several participants suggested simplifying specific interface elements, most notably the strict-versus-lenient evaluation controls and the detailed A1-B4 criteria views, which some found confusing or unnecessarily complex (4 respondents). However, the majority stated that they did not think any features needed to be removed and instead preferred refinements or additional features rather than reductions.

### 5.4. Follow-Up Analyses

A first follow-up analysis focuses on the “River” case. In this case, one sentence from the notes was, in error, not removed during pre-processing and stated that River had autism. Since participants were instructed to make their own decision, we did not remove this case from our analysis. If participants had relied on this sentence, we would expect them to conclude “ASD” for River, which would be correct. However, ADIS suggested a wrong conclusion (No ASD). When reviewing case decisions for River (Table 10), we found that when ADIS was active, only two of six decisions were correct (33.33%), when ADIS was inactive, three out of six decisions were correct (50.00%). Having ADIS active may have encouraged acceptance of its incorrect decision. Having completed their medical degree affected the pattern, however the case counts are too small to generalize.

A second follow-up analysis focused on the use of outside tools. Participants were informed they could use outside tools, but were asked to report on them. The accuracy of those with and without outside tools was nearly the same (63.64% and 60.00 respectively) (Table 11). In addition, outside tools were used by both those who had completed their medical degree and those who had not. Of the eight cases with outside tools use, most were to review ASD related information: “Used Chat GPT to support with finding key evidence”, “DSM-5 Autism Criteria”, “Referred to DSM5 “, Looked up motor milestones, definition of Connors’ Rating Scale”, “Read UpToDate article on Autism Spectrum Disorder in children, “ and “Google to refresh my memory “. Twice it was used for writing support: “Chat GPT to summarize”.

Finally, we analyzed the specific information participants would pass on to a mental health specialist along two dimensions: response length (word count) and diagnostic framing (explicitly referencing DSM terminology, such as criteria codes A1-B4, or formal diagnostic language such as “meets criteria”).

All responses were reviewed using a combination of rule-based classification and AI-assisted thematic review. Specifically, responses were processed using ChatGPT (GPT-4o, OpenAI; accessed March 2025) and then reviewed and verified, with discrepancies resolved through discussion. Final classifications reflected the researcher’s judgment, with AI assistance serving as a preliminary analytical scaffold. This approach is consistent with emerging frameworks for AI-assisted qualitative analysis in health informatics research (33, 34).

Overall, responses were relatively brief (M = 26.44 words, SD = 31.12), and 18.3% contained DSM-focused language. Clear differences emerged by training level (Table 12). Participants who had completed medical training produced shorter responses (M = 18.57, SD = 13.57) compared to those without completed training (M = 34.48, SD = 40.75). In contrast, DSM-focused responses were more common among trained participants (27.7%) than among those without complete training (8.7%).

These findings suggest that clinically trained participants tended to communicate information more concisely and within a structured, diagnostic framework, whereas less experienced participants provided more detailed but less formally organized descriptions. This pattern is consistent with expertise-driven differences in clinical reasoning, in which experienced clinicians rely on schema-based summarization while trainees emphasize descriptive detail (35, 36).

## 6. Discussion

Overall, our results show the potential benefit of AI-supported diagnosis, especially for inexperienced clinicians. However, achieving this benefit requires high accuracy in such automated decision support tools. When ADIS suggested the correct diagnosis, participants achieved 62.5% accuracy; when ADIS was incorrect, accuracy dropped to 40.0%. This divergence is characteristic of automation bias and is not limited to mental health, but has been reported across multiple clinical domains (37) where objectivity would be expected to be easier, e.g., wound diagnostics (23). Noteworthy is also that, even though ADIS provides AI support pre-decision-making, we see the same impact as with black box systems.

Our work also demonstrated decoupling of decision confidence from accuracy. Participants were significantly more confident when ADIS was active (mean = 1.33) than when it was not (mean = 1.09; F(1,80) = 3.71, p = .058), yet confidence did not align with ADIS accuracy. This pattern is not unique to our system. Future ADIS versions will treat calibration as a core design goal and aim to align expressed certainty with actual reliability. We are adjusting the interface to show model certainty cues alongside labels, an approach shown to improve appropriate trust without sacrificing usability (30).

A significant interaction between ADIS correctness and training completion (F(1,38) = 5.049, p = .031) revealed qualitatively distinct reliance patterns. Trainees achieved 0% accuracy when ADIS was incorrect but 68.75% when it was correct. Experienced clinicians showed the reverse: 66.67% accuracy when ADIS was wrong but only 56.25% when it was right, pointing to productive skepticism that could, in some cases, become excessive. This shows that AI-powered decision support can help inexperienced clinicians make better decisions. However, there is the danger of automation bias, as argued in the literature, with trainees especially prone to uncritical acceptance of AI because they lack the clinical experience and self-confidence needed to challenge system outputs (24). Together, these findings frame the crossover pattern we observed: expertise suppresses automation bias but introduces over-reliance on one’s own diagnostic schema at the expense of valid AI signals.

The open-ended response analysis provides convergent evidence for these differential reliance patterns. Trained participants produced notably shorter responses (M = 18.57 words) compared to trainees (M = 34.48 words) and were more than three times as likely to use DSM-focused language (27.7% vs. 8.7%). This suggests that experienced clinicians approach clinical communication using established habits: they encode observations concisely, map them onto formal criteria, and filter out information they deem less relevant.

Participants rated ADIS positively overall. Most found it easy to use and agreed that others would learn it quickly. Nevertheless, a minority reported confusion about the threshold slider, the tool’s most AI-specific feature. Favorable global impressions can mask gaps in understanding of AI-specific functionality, particularly for features that require basic AI literacy about model behavior. These findings align with expert consensus that AI literacy training is a prerequisite for safe clinical AI deployment (37, 38).

## 7. Conclusion

This study provides evidence that AI-supported clinical decision tools influence diagnostic accuracy and confidence in pediatric autism screening. ADIS improved decision confidence and, when its suggestions were correct, supported accuracy. However, when ADIS was incorrect, participants, especially trainees, showed susceptibility to automation bias, accepting AI output even in the presence of contradictory clinical evidence. Confidence remained high regardless of whether AI suggestions were correct, indicating a decoupling of certainty from actual performance that poses a patient safety concern.

Several features distinguish ADIS from black-box diagnostic AI. Its line-level behavioral evidence, DSM5 criterion alignment, and adjustable threshold provide a degree of interpretability rarely found in comparable tools. Yet, as these results show, interpretability is necessary but not sufficient. The risks of automation bias and confidence inflation persist even in transparent systems, and they are especially pronounced among less experienced clinicians.

These findings have three practical implications and future directions. First, structured AI literacy training should accompany any clinical deployment of AI. Second, interface design should incorporate uncertainty communication and deliberation-prompting features to discourage passive AI acceptance. Third, the experience gap between trainees and expert clinicians in AI-assisted settings warrants specific attention in medical education, where supervised exposure to AI tools, with structured reflection on appropriate reliance, should become a standard component of clinical training.

As AI tools become increasingly integrated into pediatric care workflows, the imperative to decouple accuracy from confidence grows. The goal is not to replace clinician judgment but to augment it. This will require ongoing attention to how clinicians with varying levels of experience actually interact with AI systems, not just how those systems perform in isolation.

## Figures and Tables

**Table 1:** Study Case Overview

ADIS Active	Source	ADIS Suggested Decision	Number of Relevant ASD Behaviors	Case Code Name	Group Assignment
Yes	EHR	Correct	High	Sam	Group 1
	EHR	Correct	Medium	Lee	Group 3
	EHR	Correct	Low	Ashley	Group 2
	EHR	Wrong	Medium	River	Group 4
	ADDM	Correct	High	Bin	Group 1
	ADDM	Correct	Medium	Tyler	Group 3
	ADDM	Correct	Low	Blue	Group 4
	ADDM	Wrong	Medium	Obany	Group 1
No	EHR		High	Sam	Group 2
	EHR		Medium	Lee	Group 2
	EHR		Low	Ashley	Group 3
	EHR		Medium	River	Group 3
	ADDM		High	Bin	Group 4
	ADDM		Medium	Tyler	Group 2
	ADDM		Low	Blue	Group 1
	ADDM		Medium	Obany	Group 4

**Table 2:** Case Evaluation Questions

Question	Answer Options	Note
Please confirm that you reviewed the information for [case name]	Yes/ No	
Your decision	- Refer for autism evaluation - Do not refer for autism, continue to monitor - Do not refer for autism - Other	If ‘Other’ is selected, an additional question (free response) is shown: Please explain the reason for your decision
How confident are you in this decision?	- Very confident - Confident - Not confident - Not confident at all	
What are the reasons for your decision? Please list the evidence for your decision. You can copy and paste.		
What specific information would you pass on to a mental health specialist?		
Did you use outside sources to make your decision?	Yes/ No	If ‘Yes’ is selected, an additional question (free response) is shown: Please explain

**Table 3:** Participant Demographics

(N = 21)	Count (%)		
**Age**			
20–30	10 (48)		
31–40	7 (33)		
41–50	2 (10)		
51 and above	2 (10)		
**Gender**			
Female	18 (86)		
Male	2(9)		
Non-binary	1(5)		
**Race**			
Asian	3 (14)		
Black or African American	1 (5)		
Native Hawaiian or Other Pacific Islander	1 (5)		
White	13 (62)		
Prefer Not to Answer	1 (5)		
Other	2 (10)	Other	1 (5)
		MENA	1 (5)
**Ethnicity**			
Hispanic or Latino	5 (24)		
Not Hispanic or Latino	15 (76)		
**Completed Medical Training**			
Yes	11 (48)		
No	10 (52)		

**Table 4:** Accuracy of Decisions with ADIS active or not (N: number of decisions)

ADIS Active	Medical Degree Complete (N)	Percentage Decision Accuracy	Std. Deviation
Yes	No (20)	55.00	51.04
	Yes(22)	59.09	50.32
	Total (42)	57.14	50.08
No	No(20)	65.00	48.93
	Yes (22)	68.18	47.67
	Total (42)	66.67	47.71
Total	No (40)	60.00	49.61
	Yes (44)	63.64	48.66
	Total (84)	61.90	4885

**Table 5:** Confidence in Decisions with ADIS active or not (N: number of decisions)

ADIS Active	Medical Degree Complete (N)	Decision Confidence	Std. Deviation
Yes	No (20)	1.15	.59
	Yes (22)	1.50	.51
	Total (42)	1.33	.57
No	No (20)	0.70	.66
	Yes (22)	1.45	.60
	Total (42)	1.09	.73
Total	No (40)	0.92	.66
	Yes (44)	1.48	.55
	Total (84)	1.21	.66

**Table 6:** Accuracy of Decisions with ADIS Active (N: number of decisions)

ADIS-Correct	Medical Degree Complete (N)	Percentage Decision Accuracy	Std. Deviation
No	No (4)	00.00	0.00
	Yes (6)	66.67	5.164
	Total (10)	40.00	51.64
Yes	No (16)	68.75	47.87
	Yes (16)	56.25	51.23
	Total (32)	62.50	49.18
Total	No (20)	55.00	51.04
	Yes (22)	59.09	50.32
	Total (42)	57.14	50.08

**Table 7:** Confidence in Decisions with ADIS Active

ADIS-Correct	Medical Degree Complete (N)	Decision Confidence	Std. Deviation
No	No (4)	1.25	.50
	Yes (6)	1.50	.55
	Total (10)	1.40	.52
Yes	No (16)	1.12	.62
	Yes (16)	1.50	.52
	Total (32)	1.31	.59
Total	No (20)	1.15	.59
	Yes (22)	1.50	.51
	Total (42)	1.33	.57

**Table 8:** Usability Survey (N: Number of responses)

Respondents (N=21)	Strongly Agree (%)	Agree (%)	Neutral (%)	Disagree (%)	Strongly Disagree (%)
I think that I would use this tool frequently:	2 (9.52)	10 (47.62)	7 (33.33)	1 (4.76)	1 (4.76)
I think the tool is unnecessarily complex:	0 (0.00)	2 (9.52)	1 (4.76)	16 (76.19)	2 (9.52)
I think the tool is easy to use:	4 (19.05)	10 (47.62)	3 (14.29)	3 (14.29)	1 (4.76)
I think I would need the support of a technical person to use this tool:	0 (0.00)	2 (9.52)	1 (4.76)	9 (42.86)	9 (42.86)
I think that the tool,Äôs features are well-integrated:	2 (9.52)	14 (66.67)	4 (19.05)	0 (0.00)	1 (4.76)
I think there is too much inconsistency in this tool:	0 (0.00)	1 (4.76)	5 (23.81)	13 (61.90)	2 (9.52)
I think that most people would learn to use this tool very quickly:	6 (28.57)	13 (61.90)	1 (4.76)	1 (4.76)	0 (0.00)
I found the tool very cumbersome to use:	0 (0.00)	1 (4.76)	4 (19.05)	13 (61.90)	3 (14.29)
I feel very confident using the tool:	4 (19.05)	9 (42.86)	6 (28.57)	2 (9.52)	0 (0.00)
I needed to learn a lot of things before I could use the tool:	0 (0.00)	5 (23.81)	3 (14.29)	8 (38.10)	5 (23.81)

**Table 9:** Future Use (N: Number of Responses)

Respondents (N=21)	Yes (%)	No (%)	Not sure (%)
Would you want to download the results in pdf, txt, or doc?:	15 (71.43)	5 (23.81)	1 (4.76)
Would you want to forward the results to an email address?:	11 (52.38)	10 (47.62)	0
Would you prefer the tool to evaluate all records at your facility automatically?	15 (71.43)	6 (28.57)	0

**Table 10:** Accuracy of Decisions for case “River” with ADIS active or not (N: number of decisions)

ADIS Active	Medical Degree Complete (N)	Percentage Decision Accuracy
Yes	No (2)	0.00
	Yes (4)	50.00
	Total (6)	33.33
No	No (4)	75.00
	Yes (2)	0.00
	Total (6)	50.00
Total	No (6)	50.00
	Yes (6)	33.33
	Total (12)	41.67

**Table 11:** Accuracy of Decisions With and Without Outside Tools (N: number of responses)

Outside Tool Used	Medical Degree Complete (N)	Percentage Decision Accuracy	N
No	No (35)	60.00	35
	Yes (41)	63.41	41
	Total (76)	61.84	76
Yes	No (5)	60.00	5
	Yes (3)	66.67	3
	Total (8)	62.50	8
Total	No (40)	60.00	40
	Yes (44)	63.64	44
	Total (84)	61.90	84

**Table 12:** Response Characteristics for Open-Ended Question by Training Level (N: number of responses)

Medical Degree Complete (N)	Mean Words (SD)	Percentage DSM-Focused
Yes (47)	18.57 (13.57)	27.7
No (46)	34.48 (40.75)	8.7

## Data Availability

The datasets generated and/or analyzed during the current study contain human participant information and are not publicly available due to privacy and ethical restrictions, including the risk of breaching participant confidentiality. At the time of data collection, participants were informed that their data would not be shared for future research, and the consent materials do not permit open data sharing. Data may be made available from the corresponding author on reasonable request, and any such request will be evaluated in light of the original participant consent and applicable institutional and ethical regulations. The cases used in the study are not publicly available due to the ADDM Network Data Confidentiality and Security Agreement and the EHRs’ limitation of data to university researchers. ADIS is publicly available at https://autismsupport.healthdifferential.com/. In case of updates, a link to the most recent version, if necessary, will be made available via the corresponding author’s website.

## Availability of data and materials:

Study data will be made available upon request. ADIS, in its optimized format, will be made available online.
